# Occurrence of Morphological and Anatomical Adaptive Traits in Young and Adult Plants of the Rare Mediterranean Cliff Species *Primula palinuri* Petagna

**DOI:** 10.1100/2012/471814

**Published:** 2012-04-30

**Authors:** Veronica De Micco, Giovanna Aronne

**Affiliations:** Laboratorio di Botanica ed Ecologia Riproduttiva, Dipartimento di Arboricoltura, Botanica e Patologia Vegetale, Università degli Studi di Napoli Federico II, Viale Università 100, 80055 Portici, Italy

## Abstract

Cliffs worldwide are known to be reservoirs of relict biodiversity. Despite the presence of harsh abiotic conditions, large endemic floras live in such environments. *Primula palinuri* Petagna is a rare endemic plant species, surviving on cliff sites along a few kilometres of the Tyrrhenian coast in southern Italy. This species is declared at risk of extinction due to human impact on the coastal areas in question. Population surveys have shown that most of the plants are old individuals, while seedlings and plants at early stages of development are rare. We followed the growth of *P. palinuri* plants from seed germination to the adult phase and analysed the morphoanatomical traits of plants at all stages of development. Our results showed that the pressure of cliff environmental factors has been selected for seasonal habitus and structural adaptive traits in this species. The main morphoanatomical modifications are suberized cell layers and accumulation of phenolic compounds in cell structures. These features are strictly related to regulation of water uptake and storage as well as defence from predation. However, we found them well established only in adult plants and not in juvenile individuals. These findings contribute to explain the rare recruitment of the present relict populations, identifying some of the biological traits which result in species vulnerability.

## 1. Introduction 

Few ecological studies have been carried out on plants living on vertical cliffs. However, it is repeatedly reported that these habitats act as reservoirs of relict biodiversity because they are refuges from competition, predation, fire, human activities, or climatic change in the surrounding landscape [1].

Throughout the world, cliffs have large endemic floras because species are subjected to intense selective pressure by the extreme abiotic conditions [2]. The concentration of rare species in cliff habitats reflects their intolerance of competition and disturbance and their adaptation to a harsh physical environment. These species show very slow growth and sporadic seedling recruitment. Their survival strategies are based on high genetic variability of the populations and extreme longevity of single individuals [1, 3]. These features allow cliff species to deal with the different sets of environmental factors which are distinctive of each microsite in a cliff. Therefore, conservation of these rare species is strictly dependent on the long-term maintenance of the fragile as well as stable equilibrium of surrounding abiotic conditions [1]. Consequently, environmental changes constrain the survival of single plants and start the process of consecutive decline of individuals within the population.

The conservation of rare plants cannot be guaranteed solely by their preservation through national parks and protected areas: any strategies for plant conservation need to be based on the real knowledge of the life cycle of single species and of possible interactions with other organisms, both invasive plant species and animals. The “endangered status” is worsened by continuous reproductive failure, given that sexual reproduction is the sole natural system able to improve genetic variability which is basic for the adaptation process to changing environmental conditions [4].

Completion of the reproductive cycle and high reproductive success (i.e., a large number of viable seeds produced per plant) is no guarantee of species survival in the long term. Even allowing for the accomplishment of seed dispersal and germination, seedling establishment and survival during the early stages of plant development are considered delicate phases in the regeneration process by sexual reproduction [5]. These early developmental phases can be constrained by many ecological factors of the seed microsite which can trigger phenomena of adaptation [6, 7]. Adaptation at seedling level seems to involve various strategic tradeoffs that restrict a species to optimal performance at the narrow range of the successional gradient, thus affecting community structure and dynamics [8]. Seedling survival depends on their ability to cope with numerous environmental factors such as water availability, temperature, radiation, pathogens, herbivory, and competitive interactions [9]. Space limitation due to restricted availability of microsites suitable for growth is a further constraint for cliff recruitment [1]. In a number of species of various environments, adaptive strategies for seedling survival have been shown to be based on morphoanatomical modifications aiming to regulate water relations, to protect from high levels of radiation and defend from biotic factors [7, 10–12].

*Primula palinuri* Petagna is a rare endemic species, found only in a few restricted populations on cliff sites along the Tyrrhenian coast of southern Italy. It has been inserted as Near Threatened species in the IUCN Red List; human impact, fire, competition with invasive alien species and landslides are the main threats to the species [13].

Although it is the symbol of the National Park of Cilento and Diano Valley, little is known of its biology and there is no management plan for the areas where it grows. Our preliminary studies evidenced that the species is able to form viable seeds; moreover, seedling establishment under controlled conditions is enhanced by the development of hypocotyl hairs which have a role in the control of water uptake and mechanical support [14]. However, our preliminary field surveys evidenced that the remnant populations have a structure based on old individuals, while seedlings and small plants at early stages of development are rare. Based on these considerations, the aim of this study was to monitor the Morphoanatomical development of *P. palinuri* plants from seed germination up to their adult phase in order to look for any trait that would explain the fragility of plants at early stages of development as opposed to the high survival capability of adult plants under the environmental constraints of Mediterranean cliffs.

## 2. Materials and Methods

### 2.1. Study Sites and Plant Material

Observations of plants of *Primula palinuri* Petagna were performed on populations at the Cilento National Park, along the Tyrrhenian coast of southern Italy. The species is mainly found on limestone coastal cliffs, in soil pockets inside rock fractures. The area has a Mediterranean climate [15, 16] with an annual rainfall of about 700 mm. Precipitation is concentrated in autumn and winter, while a dry period occurs from May to September.

Phenology was monitored in the field on adult plants. The presence of seedlings in the surroundings of the adult individuals was also checked every other week throughout the years 2010-2011. Plants of different ages were sampled in the field, for anatomical analyses, during vegetative growth and the summer rest. Rhizome yearly growth was measured in the field on 30 plants from three different populations. Despite the large quantity of seeds produced by the plants in all populations, no seedlings and only a limited number of young plants (1–5 years old) were found in the field. To increase the number of specimens to be used for Morphoanatomical analyses, we sampled seeds in the field and grew new plants under controlled conditions. To measure germination percent, 100 seeds from two provenances were mixed, placed in petri dishes (layered with three sheets of filter paper moistened with distilled water) and kept in the dark at 15°C. The test was replicated three times. Germinated seeds were transplanted into 0.5 dm^3^pots with a mix of peat and field soil. Plants were grown from November 2010 to June 2011 under the natural conditions of light and temperature, while they were watered daily to completely replace the water lost by transpiration.

### 2.2. Microscopy

In order to analyse the morphofunctional traits of the different vegetative organs, we dissected samples of leaves, roots, and rhizome from adult plants as well as the epicotyl, hypocotyl, and roots from plants at early stages of development. Samples were fixed in FAA (40% formaldehyde : glacial aceticacid : 50% ethanol—5 : 5 : 90 by volume) for subsequent microscopy analyses.

After fixation in FAA for several days, samples were dehydrated in an ethanol series, embedded in the acrylic resin JB4 (Polysciences, Warrington, PA, USA) and sectioned through a rotative microtome to obtain both cross and longitudinal semithin sections (5 mm). The sections were stained with 0.5% toluidine blue [17] and mounted with Canadian balsam; unstained sections were mounted with mineral oil for fluorescence microscopy. Stained sections were observed under a transmitted light microscope (BX60, Olympus, Germany), while unstained sections were analyzed under the same microscope equipped with a mercury lamp, band pass filter BP 330–385, dichromatic mirror >400 nm, and barrier filter >420 nm. Epifluorescence mode allowed us to investigate the presence of suberized and/or lignified structures as well as simple phenolics that are autofluorescent at these wavelengths [18, 19]. Photomicrographs at different magnifications were obtained with a digital camera (CAMEDIA C4040, Olympus).

## 3. Results

**Figure 1:**
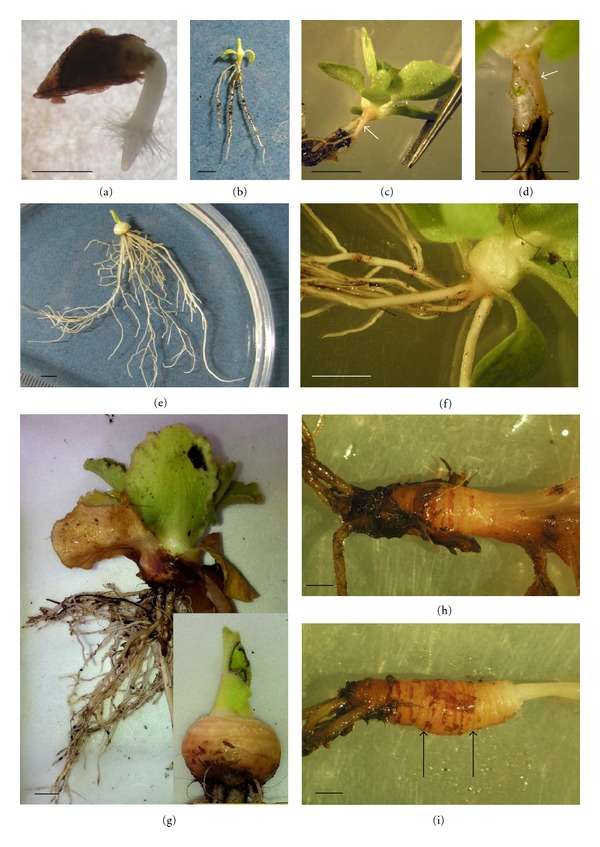
Developmental stages of *P. palinuri* plants: (a) germinated seeds with emerging root, hypocotyl ring hairs and hypocotyl; (b)–(d) very young plants at 5–7-leaf stage showing the hypocotyl enlarging into a cone in continuity with the primary root ((c), (d) arrows) and vigorous adventitious roots adjacent to the cone; (e)–(g) very young plants at 10-leaf stage showing the hypocotyl expanded into a round organ with numerous adventitious roots emerging from the base; (h)-(i) young plants with elongated hypocotyl becoming a rhizome; arrows show constrictions marking the probable transition between subsequent years/periods of growth. Bars are 1 mm in a and 0.5 cm in (b)–(i).

**Figure 2:**
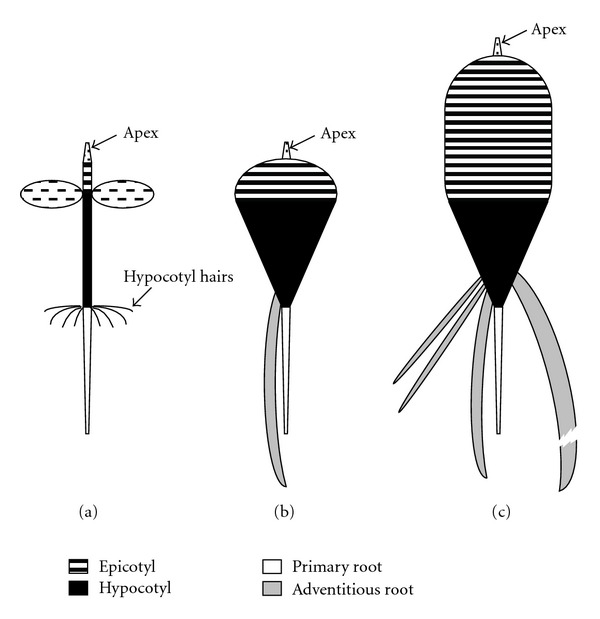
Schematic representation of the development of *P. palinuri* plants from seedling stage up to the beginning of rhizome formation: (a) seedling; (b) very young plants with hypocotyl elongating into a small cone and tuberizing epicotyl; (c) young plant with tuberized epicotyl elongating to form a rhizome and many adventitious roots emerging at the base of the hypocotyl cone.

**Figure 3:**
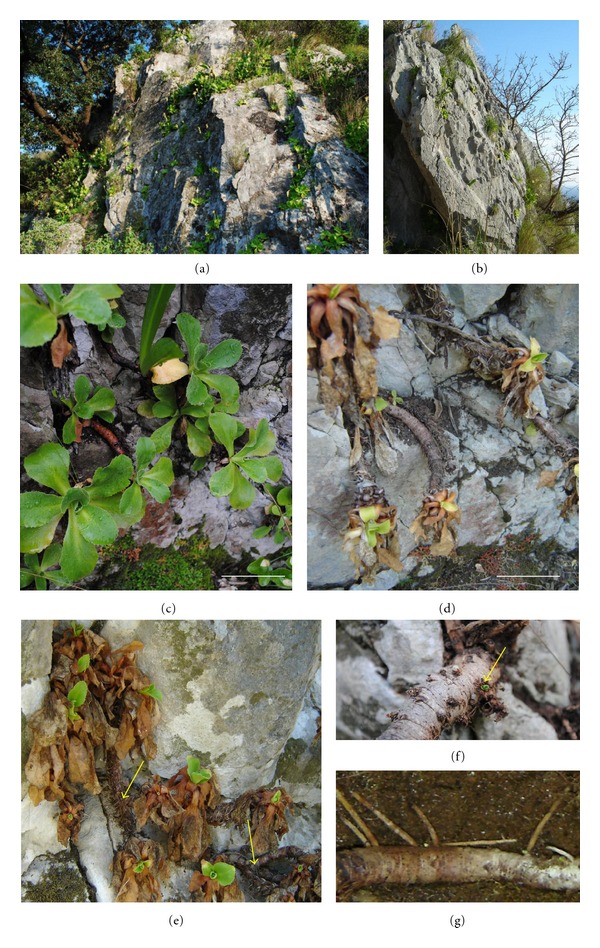
Adult plants of *P. palinuri*: (a)-(b) view of plants growing on the cliffs; (c) plants in the winter *habitus*; (d) plants in the summer *habitus* with wilting and shedding leaves; (e) branched rhizome of a single plant developing upside down; (f) rhizome with bursting lateral bud (arrow); (g) adventitious roots emerging from the rhizome and acting as tie-rods. Bars are 5 cm in (c)-(d).

*P. palinuri* develops highly viable seeds: germination percent was 86.17 (SD ± 6.14). After germination, seedling establishment is facilitated by the development of a ring of hypocotyl hairs in the region between the hypocotyl and radicle (Figures 1(a) and 2(a)). Plant growth continues through the development of tissues in the epicotyl zone, while the hypocotyl enlarges into a small cone in continuity with the primary root (Figures 1(b)–1(d), 2(b)). At this stage (5–7 leaves, 9–12 weeks) the root system is also made up by 1–3 adventitious roots at times more vigorous than the primary root (Figure 1(d)). At the 10-leaf stage (15 weeks) the epicotyl expands into a round shape, while many adventitious roots develop from its base (Figures 1(e)– 1(f)). Plant growth continues with the hypocotyl elongation that marks the beginning of rhizome formation (Figures 1(g)–1(i), 2(c)). The rhizomes of two small, young plants found in the field are reported to show subsequent growth (Figures 1(h), 1(i)). The rhizome develops indefinitely with a seasonal rhythm: vegetative growth starts at the beginning of autumn, continues throughout the winter and stops at the end of spring. New leaves develop together with rhizome elongation; they all dry up in summer with the exception of a few small apical leaves. As a consequence, plants assume two different habitus during the year (Figure 3). Growing rhizomes gradually bend due to gravity and lie on the substrate. Long rhizomes usually hang on the steep or vertical cliff-faces and develop upside down (Figures 3(c)–3(e)). Leaves are oriented independently of rhizome position. Lateral buds can burst and start the rhizome branching (Figures 3(e)-3(f)). Adventitious roots can emerge from the rhizome; frequently there are thick roots acting as tie-rods (Figure 3(g)).

The overall morphological analysis allowed to differentiate four different growth stages in *P. palinuri*: (a) seedling, immediately after seed germination until the development of the true leaves; (b) very young plants (about 1–24 months) with hypocotyl elongating into a small cone and a round tuberizing epicotyl; (c) young plant (about 2–5 years), with tuberized epicotyl elongating and enlarging to form a rhizome; (d) adult plant with long, large, and often branched rhizome.

Long rhizomes are either covered by dry leaves or naked. In the first case, lower parts of the inflorescence peduncles persist and mark yearly growth. In the second case a round, tight scar pinpoints the beginning of the new growth. Biometric measurements of these subsequent growth intervals in the field showed that rhizomes of adult plants elongate on average only 0.83 cm (SE ± 0.25) every growing season. Sometimes they break due to wind, gravity or grazing, and new rhizomes develop from basal buds. As a consequence, even if apparently small, most of the plants in the field are several tens of years old.

In order to explain why the transition from the seedling to the adult stage is a critical point in the life cycle of *P. palinuri*, we finely scanned and compared anatomical characteristics of plant organs of different ages. We analysed seedlings from seeds germinated in the laboratory. For very young plants and young plants, we used individuals both collected in the field and developed in the laboratory.

**Figure 4:**
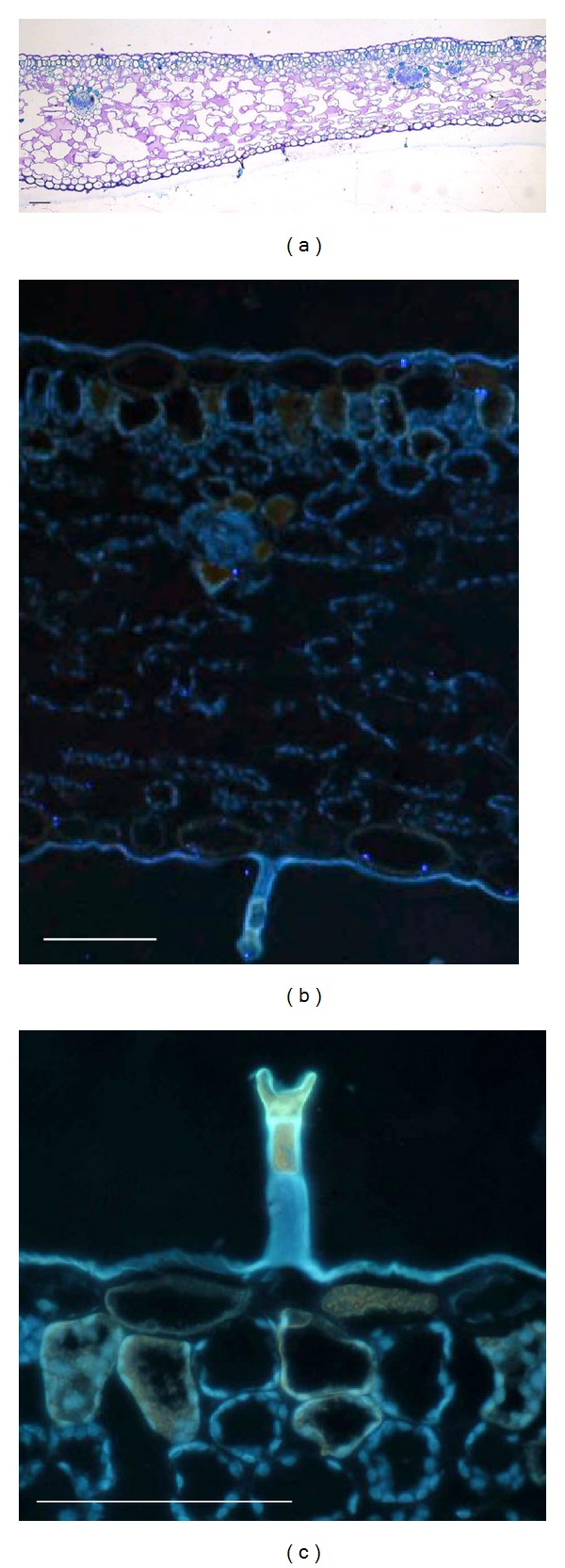
Photomicrograph of cross-sections of *P. palinuri* leaves viewed through light (a), epifluorescence (b) and (c) microscopy. UV-microscopy evidenced phenolic compounds linked to the chloroplast membranes (blue fluorescence) and filling some cells in the epidermis, palisade parenchyma and spongy parenchyma around vascular bundles (yellow-orange fluorescence). Bars are 50 *μ*m.

As regards leaves, although their size was dependent on plant age or time of season, their anatomical structure was comparable in all of them. Leaf cross-sections showed a dorsiventral structure, made of abaxial and adaxial epidermis characterised by large cells and covered by a thin cuticle, a mesophyll made of a 1-2 layered palisade parenchyma with not very elongated cells and a thick spongy parenchyma with many wide intercellular spaces (Figure 4(a)). The leaf lamina was flat, and stomata, mainly present on the adaxial surface, were well exposed. The whole structure was not dense, and a few glandular trichomes were present on both surfaces. Epifluorescence microscopy showed the presence of phenolic compounds both linked to the chloroplast membranes and filling some cells in the epidermis, palisade parenchyma, and spongy parenchyma around vascular bundles (Figures 4(b) and 4(c)). In general, leaves from both young and old plants do not survive the dry season and do not develop any anatomical trait of xeromorphy.

**Figure 5:**
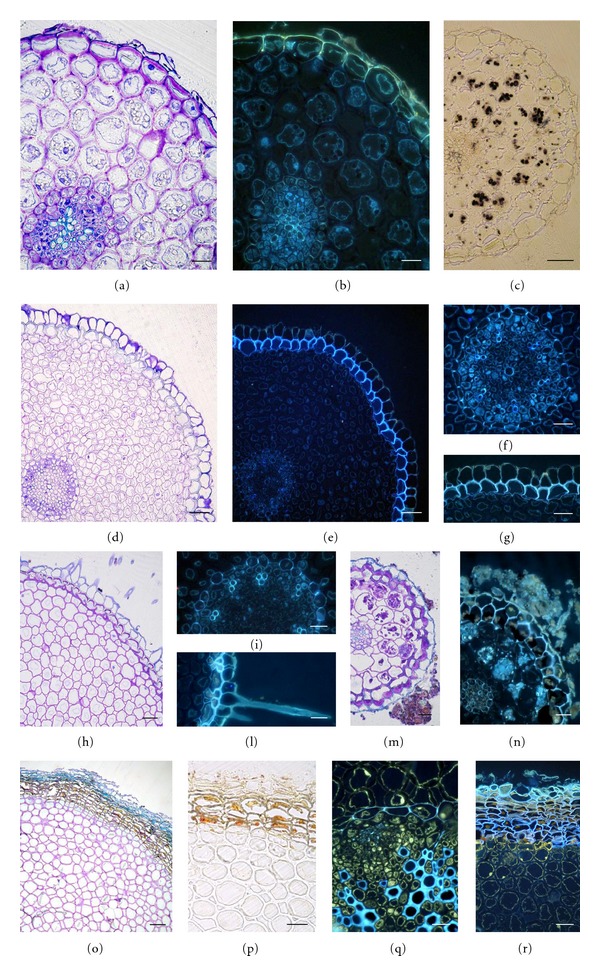
Photomicrographs of root cross-sections of *P. palinuri* plants developed under controlled conditions (a)–(g) or field-grown (h)–(r) and viewed under light and epifluorescence microscopy: (a)–(c) primary root of a very young plant; (b)–(g) adventitious root of a very young plant; (h)–(n) lateral roots from rhizomes of adult plants; (o)–(r) tie-rod roots from rhizomes of adult plants. UV-microscopy evidenced the blue fluorescence of endodermal Casparian bands (f), (i) and (q) of exodermal cell walls (e), (g), (n), and of subepidermal suberized layers (r). Phenolic compounds are evidenced by the yellow fluorescence (q) and (r). Bars are 20 *μ*m in (a), (b), (f), (g), (i), (l)–(n), and (q), 50 *μ*m in (c)–(e), (h), (p), (r), and 100 *μ*m in (o).

Roots with a diameter up to 2 mm, regardless of type (primary or adventitious roots) and growth conditions (field or laboratory), show a similar anatomical structure (Figures 5(a)–5(n)). The stele shows a poorly developed xylem surrounded by a well-developed endodermis with Casparian bands (Figures 5(b), 5(f), 5(i), and 5(n)). The cortical parenchyma consists of many layers of cells with thickened walls; the latter are not autofluorescent under UV-microscopy, suggesting that neither lignin nor suberin is encrusting them (Figures 5(b), 5(e), 5(i), and 5(n)). A suberized exodermis is present under the rhizoderm is also in the absorption zone, as testified by the presence of suberized hairs (Figures 5(b), 5(e), 5(g), 5(l), and 5(n)). The cortical parenchyma is deputed to the accumulation of water and starch.

Tie-rod roots differ from the other root types in having a more developed xylem (evaluated by comparing different root types of similar diameters), and many subepidermal layers of cells with suberized walls (Figures 5(o)–5(r)). Moreover, cortical parenchyma cells have very thickened walls and accumulate yellow-fluorescent phenolic compounds related to membranes (Figure 5(r)). Phenolics also fill the cells around the xylem and those of the subepidermal layers (Figures 5(q) and 5(r)).

**Figure 6:**
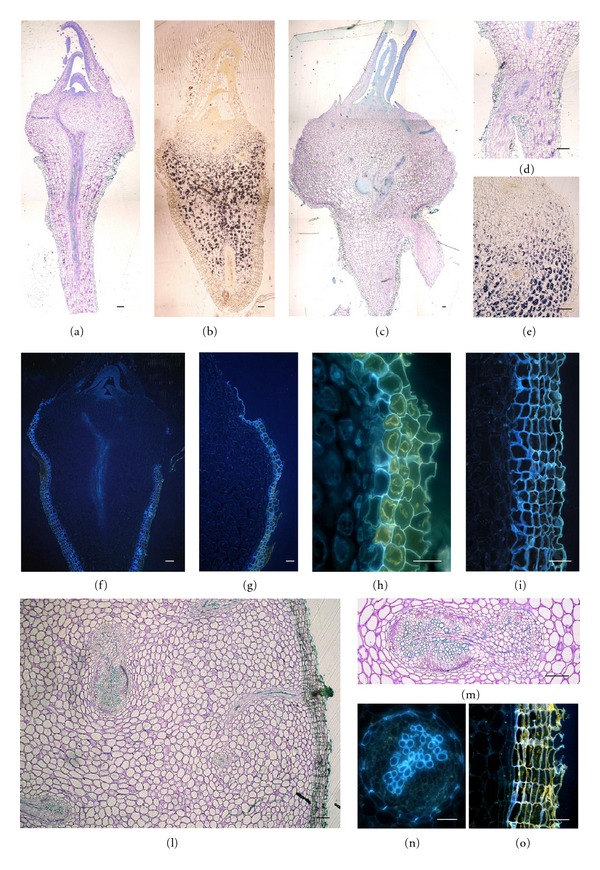
Photomicrographs of longitudinal sections of very young *P. palinuri* plants developed in controlled conditions (a)–(i) and of cross-sections of the rhizome of young and adult plants collected in the fields (l)–(o) viewed under light and epifluorescence microscopy. Subepidermal suberized layers (blue fluorescence) increasing in number if moving towards the below-ground region of the very young plants (f) and (g); endodermal Casparian bands in the stele of rhizome (n) and suberized subepidermal layers at the periphery of the rhizome (o). (h) hypocotyl; (i) root; (m) periphloematic vascular bundle. Bars are 10 *μ*m in (a)–(f), (l), (m), and 50 *μ*m in (h), (i), (n), and (o).

Anatomical analysis of entire very young plants through light and epifluorescence microscopy showed that in moving from the above-ground towards the below-ground region there is a gradient of increasing starch accumulation and the gradual appearance of structures favouring water saving from simple cuticles up to many layers of suberized subepidermal cells (Figures 6(a)–6(g)). More specifically, the occurrence of 2-3 layers of suberized cells started at the periphery of the hypocotyl zone (Figures 6(f)–6(h)); the thickness of this suberized layer increases towards the root zone (Figures 6(f), 6(g), and 6(i)).

The rhizome of adult plants is characterised by a homogeneous parenchyma tissue accumulating water and starch; many steles, often interconnected, are dispersed throughout this ground tissue of parenchyma cells (Figures 6(l), and 6(m)). Each stele has a periphloematic vascular bundle and is surrounded by an endodermis with Casparian bands (Figure 6(n)). The periphery of the rhizome contains many layers of subepidermal suberized cells which accumulate phenolic compounds (Figure 6(o)).

## 4. Discussion

Cliffs are ecosystems which are subjected to harsh physical conditions that limit the normal development of individual plants. Nevertheless, it is reported that these habitats are also characterised by high frequency of species intolerant of competition and biotic disturbance [1].

The success of seedling establishment relies on many factors, including constitutive traits of the seeds, such as size and amount of reserves. It also depends on the morphofunctional features of developing seedlings which allow prompt anchoring and absorption from the substrate [5, 20]. *P. palinuri* has developed a strategy to favour seedling survival based on both the development of hypocotyl hairs, which enhances water absorption and mechanical support, and the accumulation of phenolic compounds acting as feeding deterrents against animal predation [14]. Our field observations showed that seedling survival is a critical phase in the life cycle of this species: the low survival capability of plants at early stages of development may increase the extinction risk of *P. palinuri*.

In Mediterranean environments, species overcome summer drought by being sclerophylls, seasonally dimorphic or summer deciduous, depending on whether they adopt a strategy of tolerance or avoidance [21, 22]. Our microscopy analyses showed that leaves of *P. palinuri* have no xeromorphic traits. Their structure is not designed to accumulate or save water by reducing transpiration (e.g., low mesophyll density, reduced frequency of trichomes, and presence of well-exposed stomata at the lamina surface). Hence, plants dry leaves during periods of summer drought. *P. palinuri* leaves seem to defend themselves better against predators and to be shielded from high levels of radiation due to the presence of phenolic compounds in epidermal cells and along chloroplast membranes. The role of phenolic compounds in protection against predation and in photoprotection has been described for many species populating Mediterranean ecosystems [23–26]. In this scenario, also glandular trichomes on the leaves might be involved in a plant-animal interaction. Further work should be done to verify if they release volatile or toxic compounds.

Unlike leaves, the roots and rhizomes in *P. palinuri* adult plants seem to be specially designed to accumulate and save water as well as store reserves when starch is available after photosynthesis. Tie-rod roots growing upslope are also devised to withstand tension stress thanks to the occurrence of a large amount of lignified tissue in the stele which correlates with breaking strength as reported in other species [27]. Regulation of radial water flux in both rhizome and in roots of *P. palinuri* is exerted by the presence of a suberized exodermis in juvenile structures and of many layers of subepidermal cells with suberized walls in older structures. The occurrence of suberized layers of cells at the periphery of roots is common in Mediterranean species and is considered an effective way for the regulation of the inverse flux of water that, in extreme drought conditions, could pass from the root to the soil [28]. During Mediterranean summer drought, herbivores might be tempted by the presence of authentic “underground water tanks” in the form of rhizomes: the accumulation of phenolic compounds along membranes in the parenchyma cells of the rhizome in *P. palinuri* can be considered a valuable means to protect against predation. Defence from herbivory of cliff plants is primarily provided by inaccessibility of their habitat [1]. Nevertheless, the occurrence of phenolic compounds in fresh tissues of *P. palinuri* may play a further role in limiting predation.

While perennial organs of *P. palinuri* adult plants appear as a fortress against biotic and abiotic constraints, plants at early stages of development do not seem to be so well protected. In juvenile phases, above-ground organs seem to be inadequately protected against transpiration by a thin and delicate cuticle, while below-ground organs are designed to accumulate and save water thanks to suberized subepidermal layers whose number increases with depth in the soil. However, tuberized hypocotyls and roots do not accumulate phenolic compounds, thus being easy preys of herbivores.

In light of our findings, *P. palinuri* adult plants are well adapted to living under the particular environmental conditions of the cliff microsites in the area of Cilento National Park. By contrast, Morphoanatomical traits of plants at early stages of development do not appear specialized enough to survive the environmental constraints of Mediterranean cliffs.

Transition from seedling to young plant is a critical stage for many species; some of them avoid mortality by passing through the vulnerable stages quickly [29]. This strategy cannot be applied by *P. palinuri* whose hallmark is slow in growth throughout its development. We assume that the reduced resource availability and limited protection against drought and predation of seedlings or very young plants do not permit their survival even to brief periods of unfavourable conditions. Moreover, the limited amount of water and starch stored in the tuberized epicotyl of young plants allow them to survive only to short periods of adverse conditions.

In conclusion, our analysis of the Morphoanatomical traits of juvenile and adult plants of *P. palinuri* has added knowledge about this rare species. Obtained information, concerning critical aspects of its biology, will also be useful to assess the degree of its vulnerability and draw up measures for its conservation.
